# Are Women Really Different? Comparison of Men and Women in a Sample of Forensic Psychiatric Inpatients

**DOI:** 10.3389/fpsyt.2022.857468

**Published:** 2022-03-23

**Authors:** Judith Streb, Maximilian Lutz, Manuela Dudeck, Verena Klein, Christina Maaß, Michael Fritz, Irina Franke

**Affiliations:** ^1^Department of Forensic Psychiatry and Psychotherapy, Ulm University, Ulm, Germany; ^2^Department of Forensic Psychiatry and Psychotherapy, kbo-Isar-Amper-Hospitals, Munich, Germany; ^3^Department of Forensic Psychiatry, University Hospital Rostock, Rostock, Germany; ^4^Psychiatric Services Grisons, Department of Forensic Psychiatry, Chur, Switzerland

**Keywords:** sex differences, forensic psychiatry, substance use disorder, violence, trauma

## Abstract

**Background:**

Women in detention remain a widely understudied group. Although the number of studies in women in prison has grown in the past decade, research on female forensic psychiatric inpatients has not increased, and women are in the minority in forensic psychiatry not only as patients but also as examinees. Consequently, most treatment manuals and risk assessments were developed in male samples and apply to male offenders. However, the same treatment and risk assessment rationale can be applied in male and female mentally ill offenders only if evidence shows that no relevant sex differences exist.

**Aims:**

The aim of the present study was to examine a sample of male and female forensic psychiatric inpatients with substance use disorders and to compare the socio-demographic, legal, and clinical characteristics between the sexes.

**Methods:**

The sample included 115 male and 61 female patients. All patients were in mandatory inpatient forensic psychiatry treatment according to section 64 of the German penal code.

**Results:**

We found no significant differences between men and women in terms of educational status and vocational training. However, women were more often single and less likely to be employed full time, and they reported adverse childhood experiences more often than men. Regarding clinical variables, women appeared to be less likely to have a substance use disorder due to alcohol use and had more previous psychiatric treatments than men. Male patients were significantly younger on first conviction and detention, had more criminal records and served longer total penalties than female patients. Furthermore, men committed more violent crimes and women, more narcotics-related crimes.

**Conclusions:**

The study identified sex-specific differences in forensic psychiatric patients that should be considered in the context of forensic therapy.

## Introduction

In Germany, offenders with a substance use disorder may be ordered by the courts to be placed in a forensic psychiatric hospital in accordance with section 64 of the German penal code. Placement in such a hospital presupposes that the person's tendency to excessive consumption of drugs or alcohol is seen as a contributory cause of the delinquency and that there is a risk of further significant offenses as a result of this tendency.

**Figure 1:**
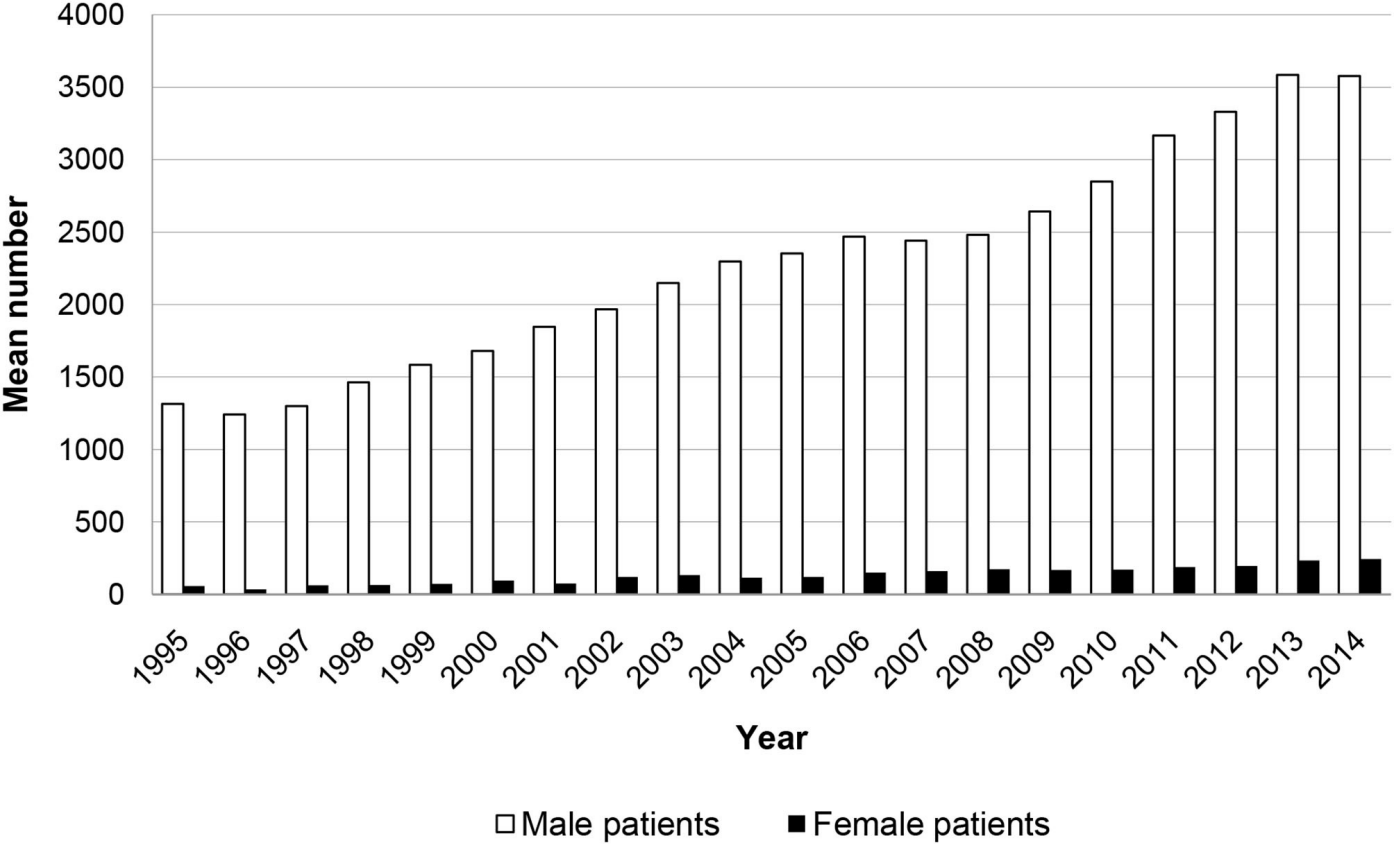
Number of male and female patients placed in mandatory drug treatment in Germany according to section 64 of the German penal code in the years 1995–2014 (30).

The number of patients in forensic psychiatric care across Germany has been increasing almost steadily in the past few decades, from 1,373 patients in 1995 to 3,822 in 2014, but women account for only 4–6% of patients (Figure 1). A lower hospitalization rate for women is found also in other Western countries, in both forensic psychiatric institutions and prisons (1).

Socio-biologists explain the higher crime burden of men in particular by differences in chromosomal structure, hormonal makeup, or innate aggressive potential. In addition, differences are attributed to sex-specific socialization. Thus, women and men are assumed to resort to different behavioral patterns for resolving conflicts and to pursue different values. Nevertheless, some authors argue that criminal behavior by women is less likely to be detected and reported, that women are less likely to be convicted and that women who are convicted are more likely to receive a lighter sentence (2).

A central question in the research literature on sex and crime focuses on the applicability of traditional criminological theories to female offenders. Some researchers reject the assumption that the same factors can explain both male and female criminality. They believe that standard theories, which are mostly male centered, overlook factors specific to female criminality. To date, the psychosocial risks of female offenders identified in mostly qualitative studies are childhood victimization (3)—which is associated with mental illness, substance abuse, depression, and anxiety (4, 5)—and extreme poverty, homelessness, and educational and vocational problems (6, 7). In turn, other findings, largely from empirical research, suggest that the same explanatory factors (e.g., educational factors, occupational skills, social ties) explain criminal behavior equally in men and women (8).

Comparing the legal characteristics of imprisoned men and women shows that women are less often convicted of violent offenses and more often convicted of property crimes or embezzlement. Violent offenses committed by women are mostly indirect or reactive and tend to occur within social relationships, whereas those committed by men tend to be antisocial, instrumental, or sexually motivated or to be due to peer pressure (9, 10). However, although men generally appear to be more physically aggressive than women with respect to arrests for violent offenses, several studies suggest that psychiatric disorders reduce the sex difference (11). For example, in forensic patients with severe mental illness (no substance use disorders), Nicholls et al. (11) found no differences in the frequency with which women and men were placed in housing because of a violent offense or in the age at placement, type of employment before placement, or previous psychiatric treatment. However, men had more criminal records and women were more likely to be in a partnership. Similarly, de Vogel et al. (12) examined sex differences in forensic psychiatry and showed that women were more likely than men to have committed homicide and arson and to have been involved in violent incidents during treatment but less likely to have committed sexual offenses. Both men and women had high rates of childhood victimization, but women were more likely to report sexual abuse. Krammer et al. (13) studied only women in forensic psychiatry and showed that two-thirds were not in a committed relationship, more than half had not completed high school, three-quarters were without a stable job before detention, nearly half grew up with an alcoholic parent, slightly more than half experienced violence or neglect in childhood, and about a quarter had been sexually abused.

The present study focuses on offenders with substance use disorder. There are some factors that are considered to be possible causes for both sexes. These include in particular addiction in the family of origin, own and family's low level of education, low income/poverty of parents, negative childhood events (such as out-of-home care, loss of an important caregiver), mental, physical, sexualized violence experiences in childhood and adolescence, prolonged performance failure, peer group as a substitute for family, lack of self-esteem and a disturbed or poorly developed gender identity (14–16). However, there are also differences in genesis between men and women. Basically, it can be said that women and girls tend to choose substances that are considered less dangerous (light cigarettes, painkillers, sleeping pills and tranquilizers, “light” alcoholic substances, such as sparkling wine, wine, beer, alcopops, cannabis), which can be consumed inconspicuously, in an adapted manner (17). Addicted women often live in stable “addictive partnerships” (about 77%), that is, the partner is also an addict. Men, on the other hand, very often live in relationships in which the partner does not have an addiction problem (only about 33% of addicted men live in so-called addiction partnerships) (14, 17). Overall, addicted women are more excluded from the social environment than men and more often abandoned by their partners. Women are disproportionately affected by experiences of prostitution and violence. Experiences of violence reinforce the feeling of helplessness and worthlessness and can lead to increased consumption of addictive substances. It can be assumed that an estimated one third of drug-addicted women regularly engage in drug prostitution (14). In addition, it is not uncommon for women to exhibit psychosomatic reaction patterns (e.g., depression, anxiety disorders, post-traumatic stress disorders, eating disorders) even before substance-related abuse behavior or a dependence disorder (18, 19). Boys usually start using legal addictive substances (tobacco, alcohol) earlier than girls. Boys usually make their first experiences with illicit drugs in the context of groups of boys of the same age with similar previous experiences. On average, men consume more often and in larger quantities than women, and this does not only apply to illicit drugs. The forms of consumption are usually harder and riskier. Consumption usually takes place in public spaces and is loud and conspicuous. It is not uncommon for consumption to be coupled with a high propensity to violence and delinquency (especially in connection with the procurement of illegal drugs). It is often a matter of demonstrating power and strength, which can be explained against the background of social role assignments (14). Other factors specific to women that can contribute to the development of addiction are gender-related experiences of powerlessness as well as a more pronounced passivity and victim attitude. Traumatization, especially through sexualized violence in childhood with continuation into adulthood, is considered a risk factor for addiction development (as already mentioned above). Compared to control groups, addicted women and men experience sexual violence significantly more often (15, 18). The proportion of women who have experienced sexual violence is higher than among addicted men. Among substance abusers, 45% of women report having suffered sexual violence before the age of 16 (compared to 16% of men) (14). Furthermore, biological differences have to be taken into account. For example, the consumption of alcohol has a more damaging effect on the female organism than on the male organism. The consequences of harmful consumption often occur earlier in women. So do the resulting sequelae, such as faster disability, negative social reactions, feelings of guilt and shame, and social isolation (17).

As mentioned above, the vast majority of research in forensic psychiatry has focused on male populations. Therefore, we do not know whether the theoretical knowledge about male offenders is sufficiently valid and useful for female offenders. Significant gaps remain in our knowledge about the importance of sex differences in, for example, the development of offending and risk factors and assessment of violence. Therefore, the present study focused on differences in socio-demographic, legal, and clinical characteristics between male and female inpatients with substance use disorders in forensic psychiatry with the aim to support the development of sex-specific theories of delinquent behavior and treatment programs, if necessary.

## Methods

### Participants

The sample included 115 male and 61 female patients. Participants were recruited from the departments of forensic psychiatry at three German hospitals (Guenzburg, *n* = 85; Taufkirchen, *n* = 55; and Rostock, *n* = 36). Slightly more than half of all patients accommodated in the three psychiatric hospitals took part in the survey (64%). The non-participating patients declined to participate or could not be approached at the time of the survey (because they were in therapy sessions or were outside the hospital). All patients were in mandatory drug treatment according to section 64 of the German criminal code. In Germany, admission to a forensic psychiatric hospital follows a court decision according to section 63 or 64 of the German criminal code. If a person has committed a serious offense as a result of a severe mental disorder, e.g., schizophrenia, and if there is a high risk of reoffending, the court orders that person's placement in a forensic psychiatric hospital according to section 63. Hospitalization according to section 64 requires a diagnosis of a substance use disorder, a high risk of reoffending, and a favorable treatment prognosis. In some respects, the living conditions in forensic psychiatric hospitals are similar to those in prisons, however there are also differences. Because forensic patients have a mental or substance use disorder, they are cared for by doctors, psychologists, and nurses and receive treatment. The treatment objectives are to reduce the risk that the patients pose to society and facilitate their reintegration into society.

The mean (SD) age was 34.11 (9.08) years in the male sample and 35.26 (10.22) years in the female sample and was not significantly different between the sexes [*t*_(174)_ = 0.765, *p* = 0.446].

### Procedures

The study was approved by the local ethics committee of Ulm University (approval no. 194/14). The participants were recruited between July 2014 and June 2016. All patients were told about the study objectives and provided written informed consent. As per institutional policy, no compensation for participation was offered. Patients completed the questionnaire in small groups in a separate room on the ward, and a research assistant was available to offer help.

### Measures

#### Assessment of Adverse Childhood Experiences

Adverse childhood experiences were assessed with the German version of the Maltreatment and Abuse Chronology of Exposure Scale [MACE, (20), German version: KERF, (21)]. This self-rating questionnaire enables a detailed retrospective assessment of traumatic childhood experiences with the following ten subscales: physical abuse (6 items), verbal abuse (4 items), non-verbal emotional abuse (5 items), sexual abuse (12 items), emotional neglect (10 items), physical neglect (6 items), witnessed physical violence toward parents (8 items), witnessed violence toward siblings (7 items), peer emotional violence (4 items), and peer physical violence (4 items). Each item can be answered with yes or no (example item from the subscale on non-verbal emotional abuse: “Did you parents lock you in a closet, storage unit, basement, garage, or other, perhaps even very cramped, dark location?”). Responses of “no” were coded as 0, and responses of “yes,” as 1. For each scale, the values were summed and transformed by linear interpolation to obtain comparable scale values. Last, a total score was calculated as the mean score of all ten scales. The authors of the instrument provide cut-off values for each subscale. Furthermore, participants had to specify at what age and for how long the adverse experience took place. For this purpose, they marked the period on a scale ranging from 1 to 18 years. In addition, the questionnaire captures clinically relevant additional information on financial stress (debt, poverty, little money) and loss of a parent (death, divorce, separation, foster care/institutional care) with separate items that are not assigned to a scale. The convergent and divergent validity of the KERF is established, as demonstrated by satisfactory associations with the Childhood Trauma Questionnaire (22) and with psychopathology scales [Hamilton Depression Scale (23), Borderline Symptom List (24), Shutdown Dissociation Scale, (25)] [see (21)].

#### Assessment of Sociodemographic, Clinical, and Legal Characteristics

Socio-demographic (school-leaving qualification, marital status, vocational training, last occupation) and clinical characteristics (ICD-10 diagnosis, previous psychiatric treatment, suicide attempts) were obtained from the patient file, and information about the index offense and previous convictions was taken from the official court records.

### Data Analyses

Data were analyzed with IBM SPSS Statistics version 28.0 (Armonk, NY: IBM Corp.). In a first step, mean values, SDs, and relative frequencies were calculated. Sample characteristics of the male and female groups were compared with Student *t* test, Mann-Whitney *U* test, Pearson's chi-squared test, and Fisher's exact test. To avoid problems due to multiple testing, the significance level per test family (i.e., socio-demographic, clinical and legal characteristics) was adjusted according to the procedure of Benjamini and Hochberg (26). An *p* < 0.05 was considered to indicate a statistically significant difference.

## Results

**Figure 2:**
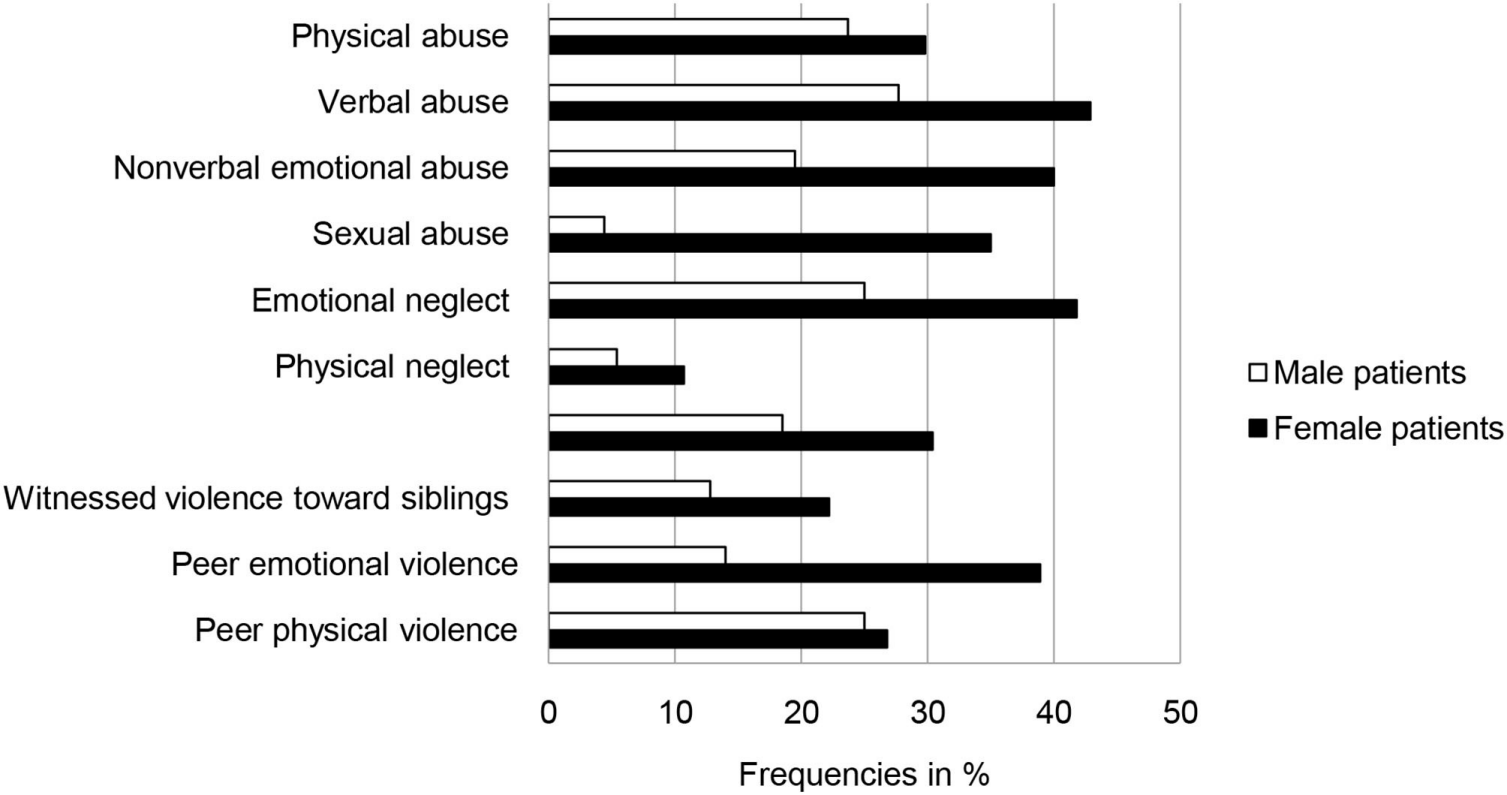
Frequencies (in percent) of adverse childhood experiences above cut-off values in male (*n* = 115) and female (*n* = 61) forensic psychiatric inpatients with substance use disorders (multiple answers possible).

Female patients were significantly more likely than males to report adverse childhood experiences (proportion of patients with scores above the reported cut-off values in at least one of the KERF subscales: female patients, *n* = 45 (79%); male patients, *n* = 60 [63%; Chi^2^_(1)_ = 4.158, *p* = 0.041, Cramer-*V* = 0.165]. Figure 2 shows the mean frequencies of each type of adverse childhood experience in men and women. Women reported significantly more adverse childhood experiences than men in the following scales: non-verbal emotional abuse [Chi^2^_(1)_ = 7.081, *p* = 0.012, Cramer-*V* = 0.223], sexual abuse [Chi^2^_(1)_ = 31.083, *p* < 0.001, Cramer-*V* = 0.458], emotional neglect [Chi^2^_(1)_ = 4.439, *p* = 0.043, Cramer-*V* = 0.176], and peer emotional violence [Chi^2^_(1)_ = 11.923, *p* < 0.001, Cramer-*V* = 0.285].

**Figure 3:**
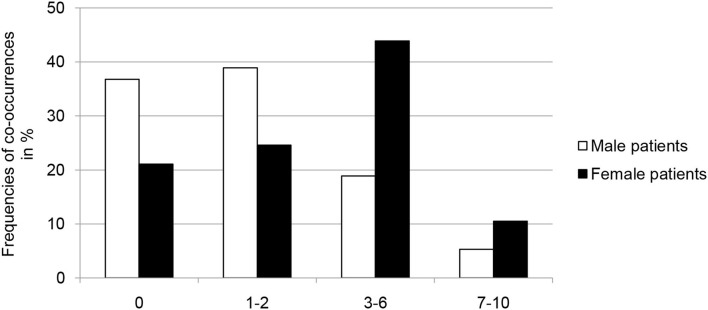
Frequencies (in percent) of co-occurrences of adverse childhood experiences in male (*n* = 115) and female (*n* = 61) forensic psychiatric inpatients with substance use disorders.

Women reported also significantly more co-occurrences of adverse childhood experiences than men [Chi^2^_(3)_ = 14.249, *p* = 0.002, Cramer-V = 0.306; Figure 3]. Further analyses showed that women had been exposed to adverse childhood experiences over a significantly longer period than men (*M*_Male_ = 5.68 years, *M*_Female_ = 8.18 years; *z* = −2.379, *p* = 0.017, *d*_Cohen_= 0.384), but the age at first adverse childhood experience was not different (*M*_Male_ = 5.75 years, *M*_Female_ = 5.31 years; *z* = −0.224, *p* = 0.823).

We found no significant differences between men and women in terms of family circumstances, highest school-leaving qualification, and vocational training (Table 1). However, women were less often single, more often had shorter, changing relationships and were more often divorced than men. Furthermore, women were less likely than men to be employed full time and more likely to be in casual employment.

**Table 1:** Frequencies of socio-demographic variables in male (*n* = 115) and female (*n* = 61) forensic psychiatric inpatients with substance use disorders.

	**Male patients**	**Female patients**	**Statistics**
	***n*** **(%)**	***n*** **(%)**	
**Family circumstances**			
Parents separated	28 (43%)	19 (49%)	Chi^2^(1) = 0.313, *p =* 0.685
Parents divorced	22 (34%)	16 (41%)	Chi^2^(1) = 0.542, *p =* 0.530
One parent deceased	16 (24%)	8 (21%)	Chi^2^(1) = 0.193, *p =* 0.811
Foster family/institutional care	17 (26%)	9 (23%)	Chi^2^(1) = 0.095, *p =* 0.819
Financial stress	21 (32%)	14 (36%)	Chi^2^(1) = 0.141, *p =* 0.831
**Highest school-leaving qualification**			
No graduation	31 (30%)	17 (29%)	Fisher's exact test = 1.694, *p =* 0.662
Graduation after 8 years of school (“Hauptschulabschluss”)	54 (52%)	27 (46%)	
Graduation after 10 years of school (“Realschulabschluss”)	14 (14%)	12 (20%)	
Graduation from high school (“Abitur”)	4 (4%)	3 (5%)	
**Marital status**			
Single	66 (64%)	27 (47%)	Fisher's exact test = 11.908, *p =* 0.010^a^ Cramer-*V =* 0.279
Married or in solid partnership	23 (22%)	12 (21%)	
Shorter, changing relationships (<6 months)	0	4 (7%)	
Divorced	13 (13%)	13 (22%)	
Widowed	1 (1%)	2 (3%)	
**Vocational training/college degree**			
Did not complete vocational training	60 (58%)	34 (59%)	Fisher's exact test = 0.175, *p* = 1.000
Completed vocational training	41 (40%)	23 (40%)	
Completed college degree	2 (2%)	1 (2%)	
**Last occupation**			
Full-time employment	29 (28%)	9 (16%)	Fisher's exact test = 19.966, *p < * 0.001^b^, Cramer-*V =* 0.361
Not working (housewife, -man)	4 (4%)	3 (5%)	
Occasional employment	7 (7%)	17 (30%)	
Registered as unemployed	54 (53%)	20 (35%)	
Retired/disability pension	3 (3%)	1 (2%)	
Other	5 (5%)	7 (12%)	

*According to Benjamini and Hochberg corrected significance level:*

a *p = 0.0111,*

b *p = 0.0056*.

Twenty one patients had comorbid diagnosis (6 post-traumatic stress disorder, 4 depression, 3 eating disorder, 3 mental retardation, 3 and attention deficit hyperactivity disorder, 2 schizophrenia). The frequency distribution does not differ between men and women (Fisher's exact test = 8.154, *p* < 0.001). The overall frequency of a comorbid personality disorder and the age at first inpatient treatment were not significantly different between male and female patients (Table 2), but sex differences were found in all other clinical variables. Women were more likely to have emotionally unstable personality disorder, while men were more likely to be diagnosed with dissocial personality disorder. Women were less likely to have a substance use disorder due to use of alcohol or cocaine, underwent significantly more previous psychiatric treatments and reported more previous suicide attempts. In terms of the legal characteristics, male patients were significantly younger on first conviction and detention, had more criminal records and served longer total penalties than female patients.

**Table 2:** Differences in psychiatric and legal characteristics between male (*n* = 115) and female (*n* = 61) forensic psychiatric inpatients with substance use disorders.

	**Male patients**	**Female patients**	**Statistics**
	***M*** **(*SD*) or *n*, (%)**	***M*** **(*SD*) or *n*, (%)**	
**Psychiatric characteristics**			
ICD-10 diagnosis: Mental and behavioral disorders due to use of			Fisher's exact test = 19.421, *p < * 0.001^a^, Cramer-*V* = 0.345
...Alcohol	33 (32%)	6 (10%)	
...Opioids	6 (6%)	6 (10%)	
...Cannabinoids	10 (10%)	3 (5%)	
...Cocaine	5 (5%)	0	
...Other stimulants, including caffeine	5 (5%)	9 (15%)	
...Multiple drug use	44 (43%)	36 (60%)	
**Personality disorders**			
No	44 (73%)	75 (73%)	Fisher's exact test = 18.294, *p < * 0.001^b^, Cramer-*V* = 0.333
Dissocial	0	10 (100%)	
Emotionally unstable	13 (22%)	5 (5%)	
Histrionic	1 (1%)	2 (1%)	
Other	2 (3%)	11 (11%)	
Age at first inpatient treatment, years	26.75 (11.94)	25.37 (10.78)	*t*_(157)_ = −0.727, *p* = 0.468
Previous psychiatric treatments	1.85 (3.21)	4.51 (7.99)	*z* = −3.032, *p =* 0.002^c^, *d*_Cohen_ =0.494
Suicide attempts	18 (18%)	19 (34%)	Chi^2^(1)= 5.189, *p* = 0.031^d^, Cramer-*V* = 0.182
**Legal characteristics**			
Age at first conviction, years	20.59 (8.83)	25.08 (9.08)	*t*_(160)_ = 3.085, *p* = 0.002^e^, *d*_Cohen_ = 0.504
Age at first detention, years	24.09 (8.87)	29.51 (9.43)	*t*_(157)_ = 3.612, *p < * 0.001^f^, *d*_Cohen_ = 0.597
Number of criminal offenses	8.90 (9.50)	5.95 (4.03)	*z* = −2.629, *p* = 0.009^g^, *d*_Cohen_ = 0.424
Total penalty, months	78.33 (56.85)	57.46 (36.64)	*t*_(155)_ = −2.819, *p* = 0.005^h^, *d*_Cohen_ = −0.413

*According to Benjamini and Hochberg corrected significance level:*

a *p = 0.0056,*

b *p = 0.0111,*

c *p = 0.0222,*

d *p = 0.0444,*

e *p = 0.0278,*

f *p = 0.0167,*

g *p = 0.0389,*

h *p = 0.0333*.

**Figure 4:**
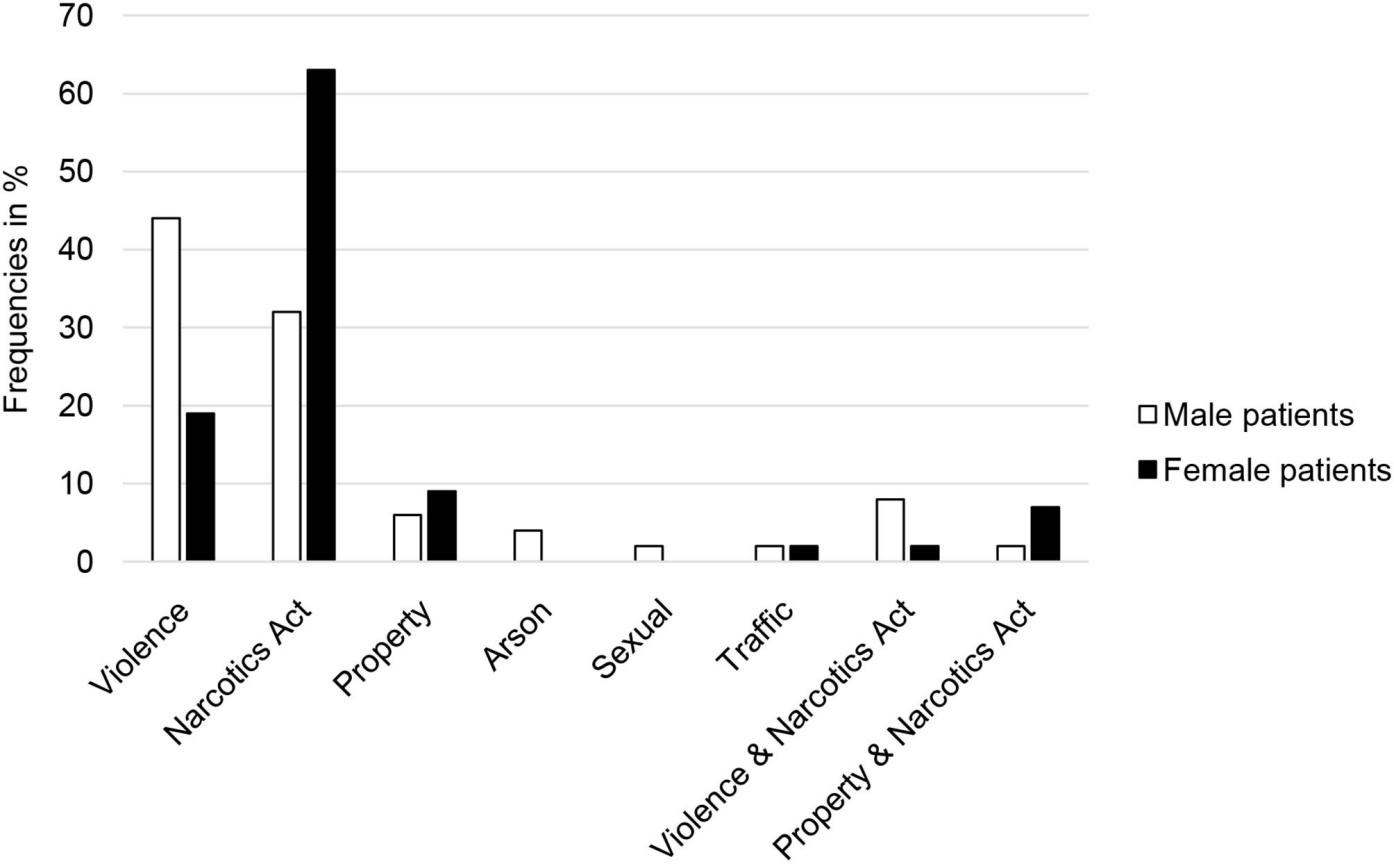
Frequencies (in percent) of the various index offenses in male (*n* = 115) and female (*n* = 64) inpatients in forensic psychiatric treatment for substance use disorders.

Figure 4 presents the frequencies of the index offense in male and female patients. The statistical analyses revealed significant differences (Fisher's exact test = 22.866, *p* < 0.001, Cramer-V = 0.383) in that men committed violent crimes more often and women, narcotics-related crimes.

## Discussion

The present study aimed to explore whether socio-demographic, legal, and clinical characteristics are different in male and female forensic psychiatric inpatients with substance use disorders. The results showed that women reported more and longer-lasting adverse childhood experiences, had more psychiatric pretreatment and later delinquency and committed fewer violent crimes. The analyses of socio-demographic characteristics showed a similar pattern to women and men in the general population, i.e., women were less likely to be single and to be employed full time.

Our findings have implications for forensic psychiatric treatment in that they support the development of sex-specific treatment programs for women that focus in particular on past trauma. Traumatized women may self-medicate by abusing drugs, which may lead to delinquent behavior; however, such behavior could be prevented if we ensure that traumatized girls and young women receive timely care and support that enables them to cope with their traumatic experiences and educates them about the negative consequences of self-medicating. Of course, this approach is relevant also in men, but the issue of trauma requires special attention in women because of the higher prevalence of victimization and the increased risk of being re-victimized (27). Furthermore, trauma related disorders, in particular complex posttraumatic stress disorders, may be underdiagnosed in forensic-psychiatric settings, because symptoms such as emotional dysregulation are misattributed to the substance use disorder or a comorbid personality disorder (12). The focus of therapeutic work with women in forensic psychiatric hospitals should be on recognizing of harmful dependency patterns and their causes and consequences in relationships, uncovering and relativizing feelings of guilt and shame in relation to social stigmatization in connection with the disease (e.g., prostitution experience), dealing with external aggression and (physical, sexual and verbal) violence, addressing education and employment as the basis of life, and as factors promoting autonomy and self-efficacy. Drug addiction among men, in turn, has another cause: the feelings of increased drive, grandiosity, and outgrowing oneself experienced in intoxication correspond to the stereotypical dynamics of masculinity. Addictive substances serve as a means of enhancing performance, experiencing risk, and exploring limits, but they are also used to deny problems, endure feelings of weakness and helplessness, and overcome fears (resulting, for example, from early childhood trauma). The consumption expectations of men also relate to the maintenance of status and power, especially through uninhibited acting out of violence. Therapeutic intervention must address these issues in the case of men and future studies should explore whether these gender-specific treatment methods result in reduced recidivism.

In addition, our findings provide further support to the many arguments for increasing the respective expertise in general psychiatry (28, 29). Substance use problems appear early in many women, who may consequently seek psychiatric help. Adequate risk prediction and risk management in general psychiatry may prevent these women from developing additional problems, committing an offense and being admitted to forensic psychiatry.

A limitation of the study is that the adverse childhood experiences were recorded retrospectively and by means of self-report. Also, so far the questionnaire has been validated only in a female sample [see (21)]. Another limitation is the unequal size of the male and female samples. Nevertheless, the study appears to identify a need for sex-specific treatment approaches for female forensic psychiatric patients with substance use disorders.

## Ethics Statement

The studies involving human participants were reviewed and approved by Ethics Review Committee of Ulm University (approval no. 143/15). The patients/participants provided their written informed consent to participate in this study.

## Author Contributions

MD and IF: designed the study. VK, CM, and IF: were responsible for administration of data collection. MF and ML: conducted the literature research. JS: wrote the first draft of the paper and conducted the statistical analysis. MD: supervised the statistical analysis and writing process. MF, ML, and IF: revised the manuscript. All authors read and approved the final version of the manuscript.

## Conflict of Interest

The authors declare that the research was conducted in the absence of any commercial or financial relationships that could be construed as a potential conflict of interest.

## Publisher's Note

All claims expressed in this article are solely those of the authors and do not necessarily represent those of their affiliated organizations, or those of the publisher, the editors and the reviewers. Any product that may be evaluated in this article, or claim that may be made by its manufacturer, is not guaranteed or endorsed by the publisher.
